# Prosthodontic Applications of Polymethyl Methacrylate (PMMA): An Update

**DOI:** 10.3390/polym12102299

**Published:** 2020-10-08

**Authors:** Muhammad Sohail Zafar

**Affiliations:** 1 Department of Restorative Dentistry, College of Dentistry, Taibah University, Al Madinah, Al Munawwarah 41311, Saudi Arabia; 2 Department of Dental Materials, Islamic International Dental College, Riphah International University, Islamabad 44000, Pakistan

**Keywords:** polymers, CAD/CAM PMMA, prosthesis, artificial teeth, dental base, prosthodontics, obturators

## Abstract

A wide range of polymers are commonly used for various applications in prosthodontics. Polymethyl methacrylate (PMMA) is commonly used for prosthetic dental applications, including the fabrication of artificial teeth, denture bases, dentures, obturators, orthodontic retainers, temporary or provisional crowns, and for the repair of dental prostheses. Additional dental applications of PMMA include occlusal splints, printed or milled casts, dies for treatment planning, and the embedding of tooth specimens for research purposes. The unique properties of PMMA, such as its low density, aesthetics, cost-effectiveness, ease of manipulation, and tailorable physical and mechanical properties, make it a suitable and popular biomaterial for these dental applications. To further improve the properties (thermal properties, water sorption, solubility, impact strength, flexural strength) of PMMA, several chemical modifications and mechanical reinforcement techniques using various types of fibers, nanoparticles, and nanotubes have been reported recently. The present article comprehensively reviews various aspects and properties of PMMA biomaterials, mainly for prosthodontic applications. In addition, recent updates and modifications to enhance the physical and mechanical properties of PMMA are also discussed.

## 1. Introduction

A wide range of polymers are commonly used for various applications in clinical dentistry [1,2,3,4,5,6,7,8]. Amongst these, poly methyl methacrylate (PMMA) is a polymer that is most commonly used in dental laboratories (to make orthodontic retainers and dentures and for repair), dental clinics (for relining dentures and temporary crowns), and industry (such as fabrication of artificial teeth) [7,9,10]. Regardless of the intended application, PMMA is conventionally available in the form of a powder–liquid system. The powder contains a clear polymer (PMMA), however additives such as pigments and nylon or acrylic synthetic fibers are added to adjust the physical properties and aesthetics to mimic oral tissues (such as gums, mucosa). The liquid component contains a monomer of methyl methacrylate, along with cross-linking agents and inhibitors [7,11,12].

PMMA gained popularity for various dental applications due to its unique properties, including its low density, aesthetics, cost-effectiveness, ease of manipulation, and tailorable physical and mechanical properties [9]. Although there are a number of concerns associated with using PMMA, such as the fracture of dentures due to water sorption and poor impact and flexural strength, the ongoing research has introduced a variety of modifications to overcome and further improve its properties (such as its conductivity, water sorption, solubility, impact and flexural strengths). For example, several studies reported the improvement of PMMA materials while reinforced using a variety of fibers [13,14,15,16,17,18,19,20,21,22], nanoparticles [23,24,25,26,27,28,29,30,31,32,33,34,35,36,37], and nanotubes [38,39,40,41,42]. Similarly, PMMA-based biocomposites with the addition of epoxy resins, polyamide, or butadiene styrene have been reported to improve the impact strength of PMMA [43]. The aim of the present article is to review various aspects of PMMA as a biomaterial for various dental applications. In addition, recent updates and modifications that have improved the material properties of PMMA are discussed.

## 2. Historical Background and Development

PMMA is an odorless polymer of acrylic acid that was reported by Redtenbacher for the first time in 1843 [44]. However, the development of PMMA for biomedical applications was a gradual process spread over a period of decades. Key stages during the development of PMMA for dental applications are shown in the Table 1. Since the 1940s, PMMA has become an essential biomaterial for dental laboratories and clinics.

Due to the acquired properties, such as the ease of processing, acceptable mechanical properties, aesthetics [55], cost-effectiveness, and relatively lower toxicity [56,57,58], PMMA has replaced previously used denture base materials (Table 2). All previously used denture base materials had certain disadvantages, and the search for an ideal denture base material is still ongoing. During the last half of 20th century, PMMA not only took over other denture base materials but also obtained remarkable popularity for the manufacturing of various dental and maxillofacial prostheses, including obturators, dentures, temporary crowns, and bridges, [50].

However, PMMA is not an ideal material due to discrepancies in its physical and mechanical characteristics. For instance, PMMA absorbs water, which may compromise its physical and mechanical properties while in use [63,64] and make it vulnerable to failure under cyclic loading [65]. Currently, a number of researchers are focused on mechanical reinforcement [30,38,51,53,66,67,68,69,70,71,72,73,74] and chemical modification [53,75,76] of PMMA to overcome its drawbacks and improve its properties, which have been reviewed in this article. In order to better understand and interpret these recent modifications, it is important to understand the chemistry, types, and properties of PMMA materials.

## 3. Chemistry and Types of PMMA

PMMA (IUPAC name: poly [1-(methoxy carbonyl)-1-methyl ethylene]) is a synthetic polymer prepared by the free radical addition and polymerization of methyl methacrylate (C_5_O_2_H_8_) to poly methyl methacrylate (C_5_O_2_H_8_)_n_ [77]. The polymerization reaction is initiated and activated by generating a free radical either chemically or with energy (such as heat, light, microwaves). In the propagation stage, the activated polymerization continues via the binding of monomers followed by termination through shifting of the free electrons to the chain end.

**Figure 1:**
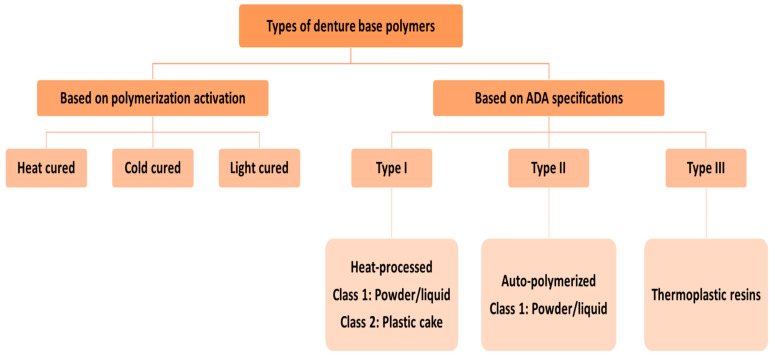
Classification of denture base polymers based on polymerization activation and according to the ADA specifications.

There are various mechanisms that can terminate the polymerization reaction, such as the addition of chemical inhibitors (hydroquinone or methyl-ether) to the monomer. Similarly, any impurities present in the monomer may inhibit the polymerization by reacting with the free radicals [78]. According to the American Dental Association (ADA) Specification No. 12 [79,80], the denture base polymers are classified into various types and classes (Figure 1). Based on the activation of the polymerization reaction, there are three main types of denture base polymers [11], which may differ from each other in terms of their polymerization reactions and compositions (Figure 1).

In addition to types I, II, and III (based on ADA specifications), the ISO 20795-1 2013 further included light-activated (type IV) and microwave-cured (type V) polymers [81]. The characteristics of various types of PMMA denture base materials are compared in the following section.

### 3.1. Heat-Cured PMMA

Heat curing PMMA materials are available in powder and liquid forms and commonly used for making denture bases and dentures [78]. PMMA powder contains PMMA, benzoyl peroxide initiator, a plasticizer (dibutyl phthalate), opacifiers (titanium and zinc oxides), fibers, and pigments or dyes. The liquid component contains methyl methacrylate (MMA) monomer, ethylene glycol dimethacrylate as a cross-linking agent, and hydroquinone as an inhibitor [10]. The polymerization reaction starts upon mixing of both components and requires heat energy (such as from a water bath) to activate the initiator. These materials contain benzoyl peroxide as an initiator, which dissociates into carbon dioxide (CO_2_), generating free radicals upon heating [82]. A high degree of polymerization results in good physical properties; however, polymerization and poor adaptation remain the main concerns [78]. Various combinations of heat curing cycles can be used for both compression and injection molding (Table 3).

The typical heat curing cycle involves a longer curing time (~9 h at 74 °C); alternatively, rapid-heat-polymerized PMMA requires a significantly shorter (20 min at 100 °C) curing time [84]. The polymerization process in the latter type is accelerated due to the presence of heat and chemically activated initiators in the composition [85]. The main purpose of the heating cycle is to achieve a high degree of polymerization and decrease residual monomers in the cured prothesis.

### 3.2. Microwave-Cured PMMA

Microwave energy is another source of heat energy that can be used to polymerize and cure PMMA. Instead of using a conventional water bath, a source of microwave energy and a non-metallic denture flask are required to polymerize these denture base materials [9]. Microwave curing has the main benefit of time effectiveness due to a short curing time (three minutes) at 500 w in a microwave [84,86], as compared to the conventional heat curing cycle, which require hours of heating followed by a cooling down period. The physical properties, including the dimensional accuracy of microwave PMMA materials, are comparable to conventional heat-cured PMMA [9]. A previous study [86] compared conventional heat-cured PMMA, injection-molded PMMA, and compression-molded and microwave-cured PMMA. It was reported that the impact and flexural strengths of injection-molded, microwave-cured PMMA were inferior to conventional heat-cured PMMA [86]. The benzoyl peroxide initiator is not present in microwave-cured PMMA, so therefore these materials cannot be cured using a conventional water bath heating cycle [86]. The main limitation of the microwave-cured PMMA materials is their weak bond strength with the acrylic teeth, which limits their prosthodontic applications [9]. In addition, the equipment and non-metallic flasks are comparatively expensive and are likely to fracture following cyclic loadings with excessive packing pressure [87].

### 3.3. Cold-Cured PMMA

Cold-cured PMMA (also known as chemically cured or auto-polymerizing PMMA) has a distinct composition and mechanism of polymerization compared to heat-cured PMMA and does not require thermal energy. A tertiary amine initiator such as dimethyl-p-toluidine [82] is added to the cold-cured PMMA, which activates the benzyl peroxide, chemically generating free radicals to initiate the polymerization [88]. However, the degree of polymerization of cold-cured PMMA is remarkably lower than heat-cured PMMA, leaving uncured residual monomers in the polymerized material, which tend to leach out [78]. Compared with heat-cured PMMA, the main advantages of cold-cured PMMA include its better dimensional stability and adaptation, resulting in minor polymerization shrinkage [78,82] however a lesser degree of polymerization [82]. However, the poor degree of polymerization compromises the mechanical properties, such as the strength and hardness [87]. In addition to monomer leaching and inferior mechanical properties, the amine initiator oxidizes with time, resulting in discoloration [82], poor color stability, and lower glass transition temperature [89]. Due to these limitations, currently cold-cured PMMA materials are only used for fabricating custom trays and provisional partial dentures and for denture repair [78]. Recently, Alqahtani [73,90] modified cold-cured PMMA to improve the mechanical properties. The addition of a hexagonal boron nitride nanopowder enhanced the elastic modulus and bending strength, while adding zirconia (ZrO_2_) increased the surface hardness. Furthermore, it was speculated that the hybrid reinforcement using ZrO_2_ and hexagonal boron nitride may remarkably improve the strength and stiffness of PMMA for durable fabrication of crowns, fixed dentures, and repairs [90]. Due to the presence of a greater amount of residual monomers and the heat resulting from exothermic polymerization, cold-cured PMMA materials are not considered for long-term oral applications.

### 3.4. Light-Cured PMMA

Light-cured (also known as visible-light-cured) PMMA works similarly to the resin-based restorative composites, which are cured when exposed to visible light [91,92]. The PMMA is modified by replacing the conventional initiator with a photo-sensitive agent (camphorquinone), which is activated and generates free radicals upon exposure to light. The light-cured PMMA materials are supplied in pre-mixed form containing PMMA fillers, silica, urethane dimethacrylate matrix, and acrylic resin monomers. To cure PMMA completely, the materials must be exposed to visible light for the required duration of time following adaptation in the cast and teeth placement. Following the polymerization, the light-cured PMMA can be finished and polished similarly to conventional heat-cured PMMA [78]. The light-cured PMMA has the advantages of easier fabrication and allowing full control over of curing, providing sufficient time for the manipulation and adaptation before initiating the polymerization [9]. Additionally, polymerization shrinkage, the existence of residual monomers, and bacterial adhesion are reduced compared to with heat- and cold-cured PMMA materials, which are the potential benefits of light-cured PMMA [93]. However, light-cured PMMA materials are not used commonly due to their drawbacks, such as their limited curing depth, technique sensitivity, and cost [87]. The mechanical properties of light-cured PMMA materials are slightly inferior to conventional PMMA [94], and therefore their applications are limited to relining and repair of denture bases [10], in addition to fabrication of custom trays and base plates for complete dentures. It is obvious that each type of PMMA material differs in terms of its composition, polymerization, benefits, and drawbacks. Accordingly, no single type can be labelled as the superior material for all dental applications. Therefore, dental teams should consider the characteristics and suitability of each type of PMMA when selecting a material for a specific application.

## 4. Manipulation

**Figure 2:**
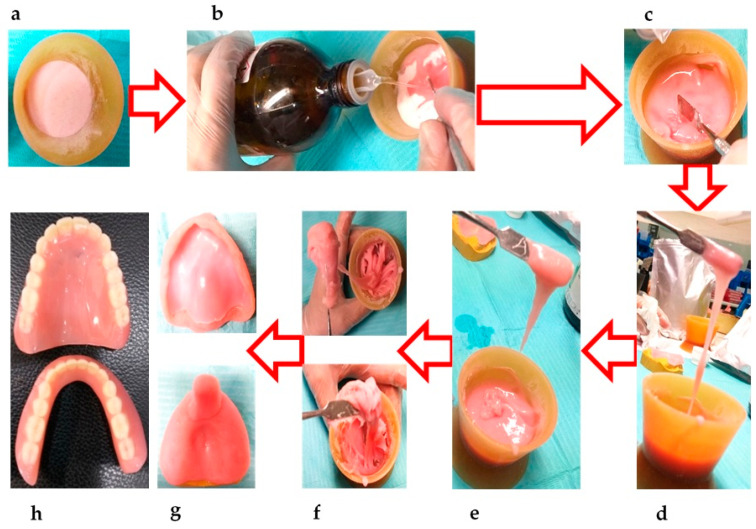
Manipulation and various stages of PMMA manual mixing and handling for denture fabrication: (**a**) PMMA powder in a mixing bowl; (**b**) pouring of the monomer solution into PMMA for mixing; (**c**) sandy stage, whereby the monomer solution has full wetted and saturated the PMMA particles; (**d**) start of stringy stage; (**e**) progression of the stringy stage; (**f**) dough stage ready for packing and plastic molding; (**g**) rubbery stage followed by the hardened PMMA, whereby plastic deformation is no longer possible; (**h**) fabricated denture using heat-cured PMMA and acrylic teeth after finishing and polishing.

The majority of PMMA formulations are supplied as polymeric powders and colorless monomer liquids (Figure 2a and Figure 2b, respectively). The exothermic chemical reaction starts upon mixing of the PMMA powder and liquid, which harden either chemically (cold cure) or via energy application in the case of heat-cured PMMA materials [7,11]. The typical process of PMMA polymerization (initiation, activation, propagations, and termination) is described in Section 3. To avoid any discrepancies, it is necessary to use the recommended ratio of PMMA powder and liquid (2.5:1 *w/v* or 3–3.5:1 *v/v*). In the case of a high powder-to-liquid ratio, this means that not all of the PMMA beads will be wet, leading to a granular texture, while a low powder-to-liquid ratio enhances polymerization shrinkage and dimensional changes [9]. Depending on the consistency, the manipulation of PMMA mixing can be divided into distinct stages (such as sandy, stringy, doughy, rubbery, and stiff stages), which are followed by packing and finishing of denture bases (Figure 2).

The mixing process starts with the sandy stage (Figure 2c), in which the monomer wets the PMMA granules, giving the mixture a grainy appearance. There is hardly any chemically interaction or polymerization during this stage [32]. In the stringy stage, the monomer molecules attack and dissolve PMMA particles and disperse the polymer chains in the liquid phase. At the same time, the larger PMMA particles begin to unfold and enhance the viscosity [95]. Typically, the stringy stage is characterized by formation of sticky strings upon touching or stretching (Figure 2d,e), further progresses to the doughy stage (Figure 2f). However, the mixture still contains a number of undissolved polymer chains. This stage is characterized by the loss of stringiness and stickiness [95]. In addition, the mixture in the doughy stage is considered suitable for packing into a dental flask (Figure 2f). The dough-forming time (from mixing to doughy stage) is affected by various factors, such as the polymer’s molecular weight, the particle size and surface area, temperature, the presence of a cross-linking agent or plasticizer, and the powder–liquid ratio [82]. The doughy stage progress to the rubbery stage by further conversion of monomers into polymers and evaporation of the residual monomers. This stage is characterized by the rubbery appearance of the matrix (which rebounds after releasing compressive or tensile stresses) and its inability to be packed into the compression molds. In the stiff stage, the continuation of polymerization and further evaporation of monomers results in hardening and reinforcement of mechanical properties (Figure 2g). The polymerized PMMA become dry, stiff, and resistant to plastic deformation [9]. Alternatively, PMMA powder and monomers can be mixed using ultrasonic mixing. Although the ultrasonic mixing of modified PMMA has been shown to be superior in terms of texture and packing [73], its use is limited in dental laboratories due to the requirement of additional equipment and the associated costs.

For the prosthesis fabrication, the PMMA in the dough stage is packed into specialized dental flasks under compression. Due to the method’s accuracy and cost-effectiveness, compression molding is frequently used for producing heat-cured PMMA prostheses [96,97]. The dental flask packed with PMMA is heated in a water bath under the defined time and temperature conditions (Table 3). To ensure proper polymerization, the temperature of the water bath should be increased gradually. Immersion of the dental flask directly into boiling water results in evaporation of the monomer (boiling point 100.3 °C) prior to polymerization and enhanced porosity in the cured material [88]. Alternatively, the injection molding technique can also be used to fabricate dentures, which requires the use of specially designed dental flasks with a sprue. The denture base material is injected through the sprue hole while the vent hole facilitates the escape of hot gasses [88]. Following the injection of heat-cured PMMA, the heat curing process is started. The polymerization shrinkage is compensated for by continuously injecting the PMMA during the curing process [78]. For both compression and injection molding, similar heat curing cycles can be used, as shown in Table 3. Considering the mismatch of the thermal expansion coefficients of PMMA and investment plaster, it is advised to cool down the dental flask to room temperature gradually to avoid distortion of the denture base. It is recommended to remove the flask from the water bath to allow bench cooling for half an hour, followed by immersion in tap water (15 min) prior to opening [78]. The PMMA dentures should be finished and polished before use in the oral cavity [98].

## 5. Properties of PMMA

**Figure 3:**
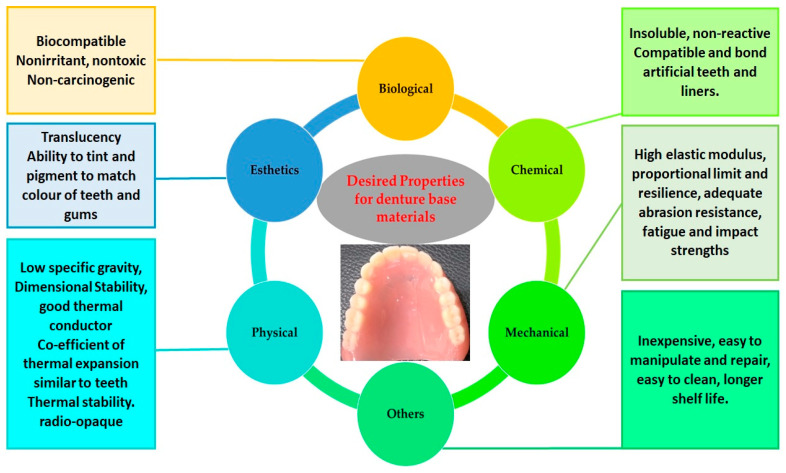
Ideal properties required for the PMMA materials for denture base applications.

The PMMA-based materials should have certain desired properties depending on the biological application. Accordingly, PMMA materials have been extensively modified and explored in relation to various chemical [77,99], biological [77,100,101,102], physical [70,77,103], and mechanical properties [66,68,70,100,103,104,105]. Primarily, it is important to understand the ideal or desired properties of PMMA materials for denture base applications (Figure 3).

Prosthodontic restorations need to be performed in complex oral environments (biofunctionality) without exerting any adverse effects on the surrounding tissues. Therefore, the PMMA used for denture base materials should be biocompatible and should not cause any irritation, toxicity, or mutagenicity to the oral tissues [78,106]. Chemically, PMMA needs to be highly insoluble in saliva and oral fluids. It should be non-reactive to nutrients, however should chemically bond to artificial teeth. Additionally, the PMMA should have good mechanical properties (such as high elastic modulus, proportional limit, resilience, fatigue strength, and impact strength) to withstand the forces of mastication without failure [78,106]. In addition, other properties such as having low specific gravity (light weight), thermal conductivity, ease of cleaning, and low cost are favorable for patient comfort [78,106]. The key properties of PMMA are presented in the Table 4 and discussed in the following sections.

### 5.1. Biological Properties of PMMA

Biocompatibility is the most important biological property, which is defined as a material’s ability to perform in a biological environment with a favorable host response [56]. Although there are very limited biocompatibility issues for properly fabricated heat-cured PMMA, the presence of uncured or residual monomers in the cured denture base has been reported to be associated with mucosal irritation [109], tissue inflammation, and cytotoxicity [110,111]. Therefore, adding a larger amount of monomer solution while mixing PMMA leaves residual monomers and results in corresponding cytotoxicity [58]. In addition to a low monomer–polymer ratio, an extended polymerization cycle also decreases the residual monomer quantity and cytotoxicity compared to short curing cycles [112]. As a comparison, heat-cured and microwave-cured PMMA eluted significantly lower amounts of monomers and cytotoxicity compared to cold-cured PMMA [101]. The immersion in water prior to use considerably reduced the residual monomer amount, as well as the cytotoxicity of PMMA [112]. In addition to biocompatibility, the excessive amount of residual monomers is also directly related to the solubility of PMMA denture base materials, as the leaching of unreacted monomers enhances the degree of solubility [113,114]. Therefore, the amount of residual monomers and the cytotoxicity of the PMMA denture base can be controlled by enhancing the degree of polymerization using heat-cured PMMA and the recommended heat curing cycle [110]. Accordingly, properly cured PMMA materials with low amounts of monomers are likely to have good biocompatibility. The volatile nature of monomers and the dusty nature of the fine PMMA particles may result in professional hazards to the dental staff who are involved in the handling of this material [115]. To reduce the concentration of monomer vapors and exposure in dental clinics and laboratories, appropriate safety measures such as the use of personal protection (mask, gloves) and airtight containers, good ventilation, and quick disposal of any spills should be practiced. In addition, dental staff should be educated about the toxic and volatile nature of monomer solution (no exposure to flames) and immediate washing of the skin if exposed to the monomer solution [115].

### 5.2. Physical Properties of PMMA

PMMA materials have favorable physical properties for denture base applications. Sorption (water or oral fluid uptake) takes place while they are immersed [116]. Due to the molecular polarity, the water molecules infiltrate through the polymer chains and act as plasticizers [117]. In addition, the penetrated water molecules lead to the expansion of the PMMA and affect the dimensional stability [64]. Another related property is the solubility, which may affect the dimensional stability [116,118]. Therefore, the sorption and associated solubility should be kept to a minimum. According to ISO 20795-1 [81], the sorption and solubility should be less than 32 and 1.6 µg/mm^3^, respectively. Several studies [119,120] have reported that the sorption and solubility of the currently available PMMA denture base materials are well below the required values required by ISO 20795-1 [81]. The sorption and solubility of heat-cured PMMA materials are lower compared to cold-cured PMMA materials [119]. Considering that the sorption and solubility are directly associated with the residual monomer quantity present in the prosthesis, heat-cured PMMA materials demonstrate a greater degree of polymerization, less residual monomers, and better physical properties compared to cold-cured PMMA.

The denture base materials should have acceptable thermal conductivity to conduct the temperature of food to the patients’ oral tissues. However, the thermal conductivity of PMMA is low (5.7 × 10^−4^ °C·cm^−1^) [82]; therefore, heat produced during the denture fabrication is dissipated slowly and results in surface crazing. The low conductivity can also affect a patient’s ability to sense the food temperature as compared to metallic denture bases, which are highly conductive compared to PMMA. Hot drinks may reach the pharynx or esophagus without being noticed and may burn the delicate mucosa [121]. To fulfill the aesthetic requirements, PMMA denture base materials should mimic the oral mucosa in terms of color, hue, and texture. Various transparent pigments can be added to PMMA to achieve provide good aesthetic properties, mimicking the patient’s natural tissues (Figure 2h). Additionally, the denture base materials should have high color stability and should not change (discolor) in the oral environment over time [122]. However, PMMA-based materials have demonstrated poor color stability and tend to change while functioning due to several factors. The release of residual monomers enhances the water sorption and discoloration due to ingress of various molecules. Other factors associated with staining and color changes of PMMA include the fabrication porosity and periodic consumption of various beverages such as coffee, tea, carbonated drinks, and alcohol [123,124]. Therefore, PMMA dentures may require replacement after a period of time due to inferior wear resistance, staining, and poor aesthetic properties.

The polymerization shrinkage (linear and volumetric) may result in remarkable dimensional changes and inaccuracies during denture fabrication [78]. Therefore, the lowest level of polymerization shrinkage is desired for dental applications. Comparing various types of PMMA, light-cured PMMA has demonstrated lower amounts of residual monomers and less polymerization shrinkage compared to heat- and cold-cured PMMA materials [93]. In terms of heat-cured PMMA, the injection molding technique has been suggested to reduce polymerization shrinkage and improve the marginal seal, as compared to conventional compression molding [96,125]. In the injection molding technique, continuous injection of the PMMA compensates for the polymerization shrinkage [78]. Additionally, various modifications of PMMA, such as reinforcement using fibers [76,126] or carbon nanotubes [41], can remarkably decrease the polymerization shrinkage and dimensional accuracy of the dental prostheses. Radiopacity is another physical property that is ideally required in restorative dental materials. Radiopaque restorative materials appear white and can easily be distinguished from tissues in diagnostic radiographs. For example, in case a broken piece of a denture is accidently swallowed, radiopaque objects can be detected easily [127,128]. Due to its polymeric nature, PMMA is a radiolucent material that is hard to detect in radiographs [129]. Inducing radiopacity by modification is challenging, as most heavy metal salts are not compatible with PMMA [7]. The incorporation of various heavy metals has been explored to enhance the radiopacity [130,131]. Although various modifications have improved the radiopacity of PMMA to some extent, there are certain concerns, such as a lack of physical or chemical binding of additives to the matrix and the salts being prone to leaching out of the denture base [132,133]. Similarly, adding heavy metal salts improved the radiopacity in one study, however various properties, including the polishability, bonding strength, and aesthetic properties, were significantly affected [133]. In contrast, Lang et al. [130] incorporated triphenylbismuth (30% *w/w*) into PMMA and reported sufficient radiopacity without compromising the mechanical and aesthetic properties. The modification of PMMA materials by adding radiopaque agents without compromising the aesthetic and mechanical properties is challenging and requires further research.

### 5.3. Mechanical Properties of PMMA

Denture base materials are exposed to complex masticatory stresses in the oral cavity. Therefore, good mechanical properties are required for the functional performance of denture base materials (biofunctionality). Considering the significance of the mechanical properties, several modifications have been reported for various mechanical properties of PMMA, including its flexural strength [14,30,33,68,134,135,136], impact strength [14,137,138,139,140], fracture toughness [141,142,143], and surface hardness [67,98,141,144,145]. The flexural strength (also known as the modulus of rupture or transverse strength) is assessed using a 3-point bending test according to the guidelines of ISO 20795-1 [81]. Ideally, the denture base should have a high flexural strength to bear the complex forces of mastication without permanent deformation or fracture [9]. A number of studies have investigated [14,30,33,68,134,135,136] flexural strength using various methods and types of PMMA. Barbrosa et al. [146] reported good flexural strength for heat-cured, cold-cured, and microwave-cured PMMA (92.84 ± 4.73, 84.40 ± 1.68, and 109.63 ± 5.31 MPa, respectively). The immersion of PMMA in water for a longer time decreased the flexural strength, resulting in increased water sorption [146]. In addition, the flexural strength of denture base PMMA can be affected by several factors, including the curing method, chemical composition, degree of polymerization, dimensions, and storage [9].

Although the flexural strength demonstrates a material’s ability to withstand tensile, compressive, and shear stresses, the fracture toughness and impact strength of PMMA are also important for denture base applications. The fracture toughness describes a material’s ability to resist crack propagation due to notches or defects present in the surface [147]. The fracture toughness of heat-cured PMMA (2.06 ± 0.17 MN/m^3/2^) is significantly greater than cold-cured PMMA (1.63 ± 0.1 MN/m^3/2^), regardless of the testing methodology [148]. Therefore, heat-cured PMMA performs better in inhibiting crack propagation and fracture. The impact strength describes the amount of impact energy required to cause a fracture [78,147]. High impact strength is desired to prevent the fracture of dentures when exposed to a high impact force, such as accidental dropping. The impact strength of a denture base can be remarkably reduced by the presence of tiny surface defects as a result of wear and tear. Even a micron-sized surface defect may act as a notch for crack propagation and fracture [149]. Certain additives, such as butadiene styrene, can significantly improve the impact strength [87], however they may affect other properties, such as the hardness and modulus of elasticity. The wear resistance of currently available PMMA materials is significantly lower compered to casting alloys and dental porcelains [150]. The wear resistance is directly related to the material’s surface hardness, which is lower in case of PMMA compared to casting alloys and dental porcelains [150,151]. Therefore, further improvements in the mechanical properties of PMMA are desired, particularly the impact strength, flexural strength, hardness, and wear resistance.

### 5.4. Chemical Properties of PMMA

Ideally, the denture base materials should be highly insert and chemically non-reactive with oral fluids and nutrients. During function, the denture base materials are exposed to a variety of nutrients, which may drastically vary in terms multiple factors, including their chemical nature, pH, and temperature [152]. Chemically, PMMA materials are organic resins that are negligibly soluble in water, however their solubility is high in organic solvents (such as ketones and esters). Similarly, alcoholic solutions act as plasticizers and may reduce the glass transition temperature. Therefore, storage or cleaning of dentures using alcoholic solutions should be avoided. Although PMMA does not chemically react with water, repetitive storage in water may result in dimensional changes or crazing due to cyclic water sorption and drying [153]. Cross-linking agents (such as ethylene glycol dimethacrylate and 1,4-butylene glycol dimethacrylate) are commonly added to PMMA. The cross-linking agents are beneficial by reducing the tendency of PMMA to solubilize in organic solvents [154] and enhance its resistance to crazing [155].

In addition, cross-linkers diminish the formation of oxygen inhibition layers and residual monomers in polymerized materials [154]. The currently available PMMA denture base materials have demonstrated satisfactory chemical stability in the oral cavity [77].

Based on the above discussion, it is evident that PMMA fulfills most of the requirements. including having good biocompatibility and physical and mechanical properties. The main concern remains the presence of residual monomers, which are released from the materials and jeopardize various properties, including the biocompatibility. Although heat-cured PMMA has demonstrated good mechanical strength and durability, fractured dentures are commonly reported in dental clinics, mainly due to poor impact strength, brittleness, and careless handling or accidental falls. Considering their good chemical stability and fracture toughness, PMMA dentures perform very well in the oral cavity for a reasonable period of time. The addition of various fibers and particles to PMMA may further reinforce its mechanical properties (Section 7). A number of shortcomings, such as the unfavourable thermal properties (low thermal conductivity or diffusivity, high coefficient of thermal expansion), brittleness, monomer leaching, and discoloration, still require improvement and further investigations. Overall, PMMA has the benefits of easy manipulation; cost-effectiveness; and good mechanical, physical, and aesthetic properties. Therefore, PMMA materials are commonly used for denture applications.

## 6. Applications of PMMA

**Figure 4:**
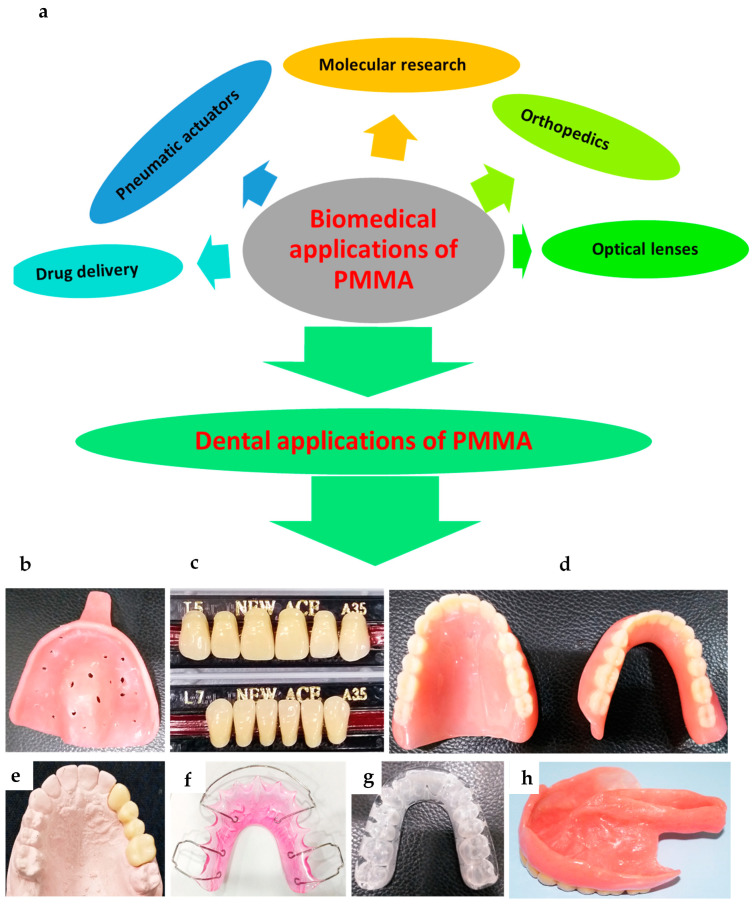
Key applications of PMMA: (**a**) in various biomedical disciplines; (**b**) secondary impression tray; (**c**) acrylic artificial teeth; (**d**) denture with acrylic teeth; (**e**) provisional fixed partial denture, crown; (**f**) orthodontic retainer; (**g**) occlusal splint; (**h**) palatal obturator replacing lost tissue following maxillectomy.

Polymeric acrylic materials are widely used for a range of applications in multiple fields, including engineering, healthcare, and dentistry (Figure 4). In addition to denture bases, other oral healthcare applications for PMMA include fabrication of artificial teeth, impression trays, temporary crowns, obturators for cleft palates, occlusal splints, printed or milled casts, dies for treatment planning, denture relining, and repair (Figure 4), which have been discussed.

### 6.1. Denture Bases, Liners, and Reliners

The alveolar bone is a dynamic tissue that undergoes continuous remodeling under physiological conditions [156,157]. The remodeling of alveolar bone alters the morphology of bone and denture bearing areas. A recent study reported a significantly greater resorption of the alveolar ridge in denture wearing patients compared to those who were edentulous but not wearing dentures [157]. The time-dependent alterations in the alveolar bone and the denture bearing tissues result in ill-fitting dentures, which then requiring relining to re-attain proper fit and stability. Accordingly, denture relining restores the vertical dimensions, retention, and stability of the old denture. Depending on the surface hardness, the lining materials are divided into hard liners such as PMMA [78,158]; and resilient liners. The resilient liners are elastic and composed of silicone elastomers [159,160,161], which act as shock absorber [161]. Both heat- and cold-cured PMMA materials are used for the relining of dentures (hard relining), using the old denture as an impression tray [10]. Heat-cured PMMA liners are usually supplied in the form of powder and liquid modified with the addition of plasticizers to reduce the glass transition temperature by to lubricating the polymer chains. Consequently, the PMMA material becomes comparatively flexible and resilient. The characteristic properties of PMMA liners are lost with time due to the leaching of plasticizers and increased stiffness. Cold-cured PMMA liners also can be used, which can allow chairside application without requiring laboratory work. However, due to their poor mechanical properties, leaching of monomers, and associated biocompatibility issues, their use is limited to temporary liners only. In contrast, heat-cured PMMA liners have demonstrated good bonding strength and wear resistance. However, the loss of plasticizers results in roughening of the surface, making the liners hard, and difficult to clean [10,162].

### 6.2. Artificial Teeth

Acrylic (PMMA) teeth are fabricated at an industrial scale using compression or injection molding techniques. Prefabricated teeth are supplied in a variety of tooth morphologies, sizes, and shades (Figure 4c). Once the artificial teeth have been matched to a patient’s natural teeth (regarding the color, shape), they are contoured to fit the denture base through chemical bonding. Acrylic teeth have excellent biocompatibility, and good physical, mechanical, and aesthetic properties. In contrast to heat-cured PMMA, acrylic teeth have high resilience and flexibility, and therefore are less brittle and easier to polish. Compared with porcelain teeth, acrylic teeth are lightweight and do not cause clicking sounds or wear of the opposing teeth. In addition, acrylic teeth are dimensionally stable and have a coefficient of thermal expansion matching that of the denture base [10]. Although acrylic teeth bond chemically with the denture base, the poor bond strength and debonding remain the main concerns [163]. A significant proportion of broken dentures (22–30%) involve debonding of acrylic teeth (mainly anterior teeth) due to having a smaller bonding surface area and the direction of functional stresses [164,165]. Various studies have investigated surface treatments in improving the adhesion of acrylic teeth. [166,167]. The application of methyl-methacrylate–based adhesives at the interface improves the bonding strength of acrylic teeth [167]. Similarly, the surface treatment of acrylic teeth surfaces by grinding and sandblasting increases the bond strength with the denture base [166]. The hardness, elastic modulus, and wear resistance of PMMA teeth are poor compared to the natural teeth or porcelain restorations, leading to rapid wear of PMMA teeth. Recently, silanized, feldspar-reinforced PMMA was compared with conventional acrylic teeth. The addition of silanized feldspar improved these properties (elastic modulus, hardness, and flexural strength) without compromising the impact strength. Similar results were reported by adding silica fillers, however the flexural strength was reduced [71].

### 6.3. Temporary Crowns and Bridges

The fabrication of ceramic or metal–ceramic crowns and fixed partial dentures involves time-consuming laboratory procedures [168,169], which may take several days to complete. Therefore, temporary restoration materials made from PMMA are commonly used to cover the prepared teeth until the fabrication of the ceramic restoration material is complete. PMMA temporary restoration materials can be prepared in significantly shorted periods of time, either chair-side using cold-cured PMMA or in the laboratory using heat-cured PMMA. Although PMMA is commonly used for temporary crowns and bridges, the rigidity and fracture toughness is not sufficient to withstand complex masticatory forces [170,171]. Because of the weak mechanical properties and abrasion resistance, the use of PMMA is restricted to temporary crowns and fixed bridges for a transitional period. As discussed earlier, heat-cured PMMA presents better properties compared to cold-cured PMMA. Furthermore, the polymerization of PMMA is an exothermic reaction that releases a magnificent amount of heat [50], which should be considered when using cold-cured PMMA in clinics. Although dentin is a good insulator of heat, the heat from the exothermic reaction may still reach and damage the underlying pulp, especially if the dentin thickness is less than 1 mm. The thermal damage can be minimized using either an air–water spray as a coolant or a refrigerated putty matrix as a heat sink [172]. Alternatively, temporary crowns and fixed dentures for vital teeth can be fabricated via indirect approaches using heat-cured PMMA, which has better mechanical and physical properties. Cold-cured PMMA restoration materials contain uncured residual monomers (3–5%) at significantly higher levels than heat-cured PMMA (0.2–0.5%). The residual monomers may release into the oral environment and potentially irritate oral tissues [173]. For this application, one of the main disadvantages is the polymerization shrinkage, which equals 5–7% volumetric shrinkage [174] linearly (~2%); this may lead to potential discrepancies in the fit of restoration materials, as well as microleakage. All such shortcomings should be considered while using PMMA for direct restorations in the oral cavity.

### 6.4. Repair of Dentures

Fractured dentures requiring repair are commonly reported in dental clinics [31,175]. The main reasons for denture fracture include the poor mechanical properties of PMMA [176] or accidental falls [165,177]. The repair of dentures is expensive and time-consuming [31]. In contrast, denture repair maintains the denture’s dimensions and aesthetic characteristics [178,179]. Various types of PMMA materials are used for denture repair [31]. Although heat-cured PMMA has better mechanical properties [175,180,181], the head curing procedure is time-consuming, with warpage of dentures due to reheating being the main concern [78,182]. Accordingly, cold-cured PMMA is favored for denture repair applications. Various modifications to PMMA materials can improve their strength for denture repair, with promising outcomes [31,68,141,142]. For example, the incorporation of ZrO_2_ nanoparticles improved the flexural strength of PMMA repair materials [29,30,31,142]. Previous studies have investigated light-cured PMMA for denture repair and reported several benefits, such as ease of manipulation, controlled polymerization time, no monomer issues, and better mechanical properties [93,183,184]. The repair strength of light-cured PMMA (40–44 MPa) is remarkably superior compared to heat-cured (21–34 MPa) and cold-cured (~13MPa) PMMA materials [185]. In addition to the types of PMMA, various surface treatments, such as airborne abrasion using alumina particles, laser treatment [186], mechanical grinding with a bur [187], and immersion in a monomer solution [188,189,190] or organic solvents [191], may affect the repair strength of dentures.

### 6.5. Obturators

An obturator is a special prosthesis that is constructed to restore lost maxillary tissues (Figure 4h) and functional capabilities (mastication, deglutition, speech, aesthetics) following a maxillectomy [192,193,194]. Despite advancements being made in materials science, there are only a few material choices for the construction of obturators [195]. PMMA materials are the most commonly used materials for obturators [194,195,196]. To overcome polymerization shrinkage, the injection molding technique has been advocated due to it providing better marginal sealing and accuracy compared to conventional compression molding [96,125]. There are certain disadvantages of PMMA obturators, such as polymerization shrinkage, difficulty with undercuts due to the material’s rigidity, or pressure sores on delicate tissues that have recently been exposed to the oral environment. Depending on the size of the defect, the PMMA obturators may become heavy. The weight can be reduced by using either a hollow design [197,198,199] or silicon core [200,201].

### 6.6. Computer-Aided Design and Manufacturing (CAD/CAM) PMMA

The CAD/CAM technologies are used for the fabrication of various ceramic restorations, including inlays, onlays, crowns, and fixed partial dentures [202]. More recently, several researchers [203,204,205,206,207,208,209,210,211] investigated the use of CAD/CAM technologies for the fabrication of PMMA dental prostheses and compared the materials’ properties and various aspects of the conventional and CAD/CAM PMMA materials. In contrast to the conventional “flask–pack–press” technique, CAD/CAM techniques commonly use rapid prototyping and milling techniques [208,210]. Although the chemistry of CAD/CAM PMMA is similar to that of conventional heat-cured PMMA, CAD/CAM PMMA shows superiority in terms of many properties, including its hardness, flexural strength, flexural modulus, and impact strength (Table 5). Both materials are equally biocompatible and without any significant differences in terms of monomer leaching [212]. The CAD/CAM PMMA mechanical properties and durability are improved compared to heat-cured PMMA (Table 5).

Interestingly, the adherence of fungal *Candida albicans* was remarkably reduced in CAD/CAM dentures [207], resulting in enhanced hydrophobicity and surface properties [205,206,207]. The increased hydrophobicity inhibits plaque accumulation on the polymer surface [213,214]. Therefore, due to the inhibition of *Candida albicans* attachment, CAD/CAM PMMA dentures may benefit patients prone to denture stomatitis [215,216,217,218,219]. Due to their superior mechanical properties, CAD/CAM PMMA fixed partial dentures (up to 4 units) can be used where long term (up to one year) temporization is needed in certain clinical situations [220].

### 6.7. Miscellaneous Applications

In addition to the above discussed applications, PMMA-based materials are frequently used in additional applications in various disciplines of clinical dentistry. In orthodontics, various removeable orthodontic appliances [221,222,223,224], such as retainers, bite planes, myofunctional appliances, bite guards, and occlusal splints, are fabricated using PMMA. The material characteristics and the fabrication process for orthodontic appliances are similar to PMMA denture bases, as described earlier; the only differences are their design and functional capacities, which depend on the application. In addition, PMMA base adhesives are used to bond orthodontic brackets [225]. In addition, PMMA is conveniently used to make secondary impression trays, modify primary trays, and copy (duplicate) dentures [162,226]. Due to its cheap cost and ease of manipulation, PMMA is frequently used to embed or fix specimens for restorative dentistry research [227,228] and for fabrication of study casts for treatment planning. Further modifications and improvements to the properties of PMMA are likely to overcome the major shortcomings of this most commonly used dentistry polymer and are likely to remarkably enhance its applications in various disciplines of clinical dentistry.

## 7. Modifications of PMMA

**Figure 5:**
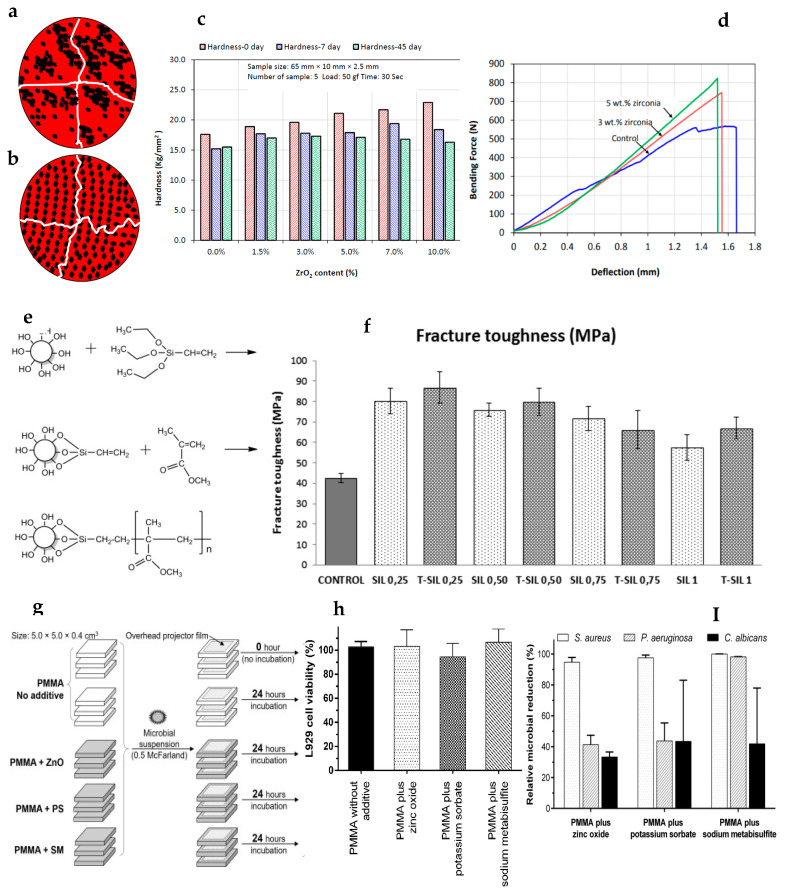
Various representative examples of PMMA modifications and associated outcomes: (**a**) randomly distributed filler particles allowing crack propagation; (**b**) uniformly dispersed particles that enhance fracture toughness through crack diversion [66]; (**c**) impregnation with ZrO_2_ resulted in increased Vickers hardness values [51] and (**d**) flexural properties [30]; (**e**) silica nanoparticle surface modification using trietoxyvinylsilane and chemical bonding to MMA to enhance the fracture toughness (**f**) of modified PMMA (from [240] with permission), PMMA (control), PMMA reinforced with silica (SiO_2_) nanoparticles measuring ~12 nm (PMMA-SIL), and PMMA containing trietoxyvinylsilane-modified SiO_2_ nanoparticles (PMMA T-SIL); (**g**) antimicrobial functionalization of PMMA by adding zinc oxide, potassium sorbate (PS), and sodium metabisulfite (SM) (from [241] with permission) showed no remarkable effects on L929 cell viability (**h**), however resulted in a significant antimicrobial action against bacteria and *Candida albicans* (**I**).

Despite there being plenty of research on and understanding of biomaterials, there is a lack of ideal biomaterials for dental applications [229]. Although PMMA certainly has many good properties (such as its strength, easy manipulation, cost-effectiveness) and has gained popularity for several dental applications, as discussed above, there are several shortcomings of this material that fall short of an ideal material’s properties for denture bases or other dental applications. For example, polymerization shrinkage (linear and volumetric) may result in remarkable dimensional changes and inaccuracies during denture fabrication [78]. Similarly, the presence of residual monomers always remains an issue affecting the properties and biocompatibility [58,115,230]. Other properties of PMMA that require improvement include its poor fatigue strength, low impact strength, weak bonding strength, low thermal conductivity, susceptibility to crazing, high thermal expansion coefficient, poor color stability, susceptibility to warpage, and porosity [231]. Each drawback has its own consequences, such as the poor flexural and impact strength resulting in fatigue failure or accidental fracture of dentures [140,165,178]. Various representative examples of PMMA modification and associated outcomes are shown in Figure 5. Therefore, the improvement of PMMA properties is required and remain a focus for researchers. In recent years, a wide range of modifications of PMMA-based materials (such as reinforcement of fibers or filler particles, antimicrobial modifications) have been investigated to improve their performance [30,31,51,52,53,68,143,232], which have been discussed.

### 7.1. Mechanical Reinforcement Using Fibers

To improve the properties of PMMA materials, a variety of fibers have been added and extensively characterized (Table 6). Several studies [13,14,15,16,17,18,19,20,21,22] have reported the improvement of various properties of fiber-reinforced PMMA. The characteristic reinforcement benefits are obtained due to the greater length of the fibers compared to the cross-sectional diameter. In addition, the enhancement of mechanical properties is affected by fibers’ morphology (length, diameter), orientation in the matrix, concentration, pre-impregnation, and silane treatment [233]. For example, the fibers can be either continuous and long, running through the span of the prosthesis [234], or can be short. Adding fibers shorter than the critical length (0.5–1.6mm for glass fibers) may negatively affect the mechanical properties [235]. Similarly, the orientation of the fibers is also important in defining their mechanical properties [235,236,237]. The unidirectional orientation of fibers results in anisotropy and reinforced strength in one direction [238], while a multidirectional or woven orientation results in isotropic dispersion of fibers and reinforced strength in all directions [239].

In addition to the fiber morphology and orientation, the fiber–matrix interface contributes greatly to the mechanical reinforcement. The pre-impregnation of fibers using monomers has been demonstrated to enhance the fibers’ properties by improving their wettability and adhesion within the matrix [250,251]. In addition, pre-impregnated fibers reduce voids in the matrix and facilitate uniform distribution of stresses [9]. Similarly, treating fibers with silane coupling agents encourages bonding between fibers and the matrix [244]. The silanated fibers demonstrated improved bonding strength compared to the untreated fibers [252,253]. However, the cost of commercially available fibers and further pre-treatment processing increase the overall cost of denture production.

### 7.2. Mechanical Reinforcement Using Particles

To reinforce the properties of PMMA materials, a variety of filler particles have been investigated, including ceramics and metals (Table 7). It is obvious from these studies that the addition of particles resulted in no biocompatibility issues and improved various properties of the PMMA, including the mechanical properties [23,24,25,26,27,28,29,30,31,32,33,34,35,36,37], thermal conductivity [23,24,31,254,255], dimensional stability [256], and antimicrobial activity [257,258,259,260,261,262,263,264,265,266], and also reduced the solubility and water sorption [267,268]. The beneficial outcomes resulting from the use of nanoparticles are achieved due to their characteristic features, such as their high surface area and better distribution [34,269].

In addition, the properties of nanoparticles (morphology, loading, type) affect the final properties of reinforced PMMA [34]. For example, The ZrO_2_ nanotubes demonstrated superior reinforcement of the mechanical properties compared to the ZrO_2_ particles [270]. Similarly, adding metallic nanoparticles enhances the thermal conductivity of the denture base and allows better judgement of food temperature [104,271]. In addition, hybrid reinforcement has been carried out using various fibers [21], particles [76,126,131,272], and combinations of fibers and particles [273]. Hybrid reinforcement improves the interface and enhance the loading capacity, improving the material’s properties [274]. Hybrid reinforcement using different types of fibers improve various properties of PMMA, including the tensile strength, flexural modulus [275], surface roughness [272], radiopacity, and thermal conductivity [276], and also results in decreased polymerization shrinkage [76,126]. Although the use of various combinations of hybrid reinforcements resulted in promising outcomes, additional research is required to explore various other materials and combinations to further improve the properties of PMMA materials.

In addition to several fibers and particles (Table 6 and Table 7), carbon nanotubes (CNTs) have been investigated to reinforce the properties of PMMA [38,39,40,41,42]. The rationale for using CNTs is their exceptional electrical and mechanical properties, the fact that they are many times stronger than steel [285], and their low density and resilience [38]. There are two distinct structurally stable types of CNTs [38]: single-walled (single seamlessly wrapped cylindrical tube) and multiwalled (array of concentric nanotubes nested concentrically) CNTs.

Wang et al. [38] added variable concentrations of multiwalled CNTs carbon to PMMA and characterized the various mechanical properties. Although adding CNTs in amounts of up to 1% improved the flexural strength and resilience, increasing the CNT concentration (2%) compromised the properties due to the insufficient dispersion of CNTs [38,42]. A similar study reported the enhancement of all tested mechanical properties form the addition of 1% of CNTs [40]. Qasim et al. added single-walled NCTs to various light-cured PMMA materials and observed no significant improvements in the flexural properties [39]. In addition to the mechanical properties, CNT loading also reduced the polymerization shrinkage remarkably [41], which is very beneficial for improving the dimensional accuracy of prostheses, particularly crowns and fixed partial dentures. Although the addition of CNTs resulted in the establishment of good interfaces, the main concern is the bad color [40,286], which limits the use of PMMA–CNT materials to non-aesthetic areas.

### 7.3. Chemical Modification of PMMA

The properties of PMMA materials can be improved through chemical modification. A typical example of chemical modification is the incorporation of rubber to form a PMMA–rubber semi-interpenetrating network, which improves the impact strength [75]. Crack propagation through the PMMA matrix is decelerated when the crack line reaches the rubber interface. Accordingly, the denture can withstand higher impact stresses before fracturing, ultimately improving the fatigue resistance. Due to the elastic nature of rubber, the denture gains more flexibility from having a reduced elastic modulus [287]. Furthermore, the properties of rubber–PMMA materials vary vastly depending on the concentration of rubber (styrene–butadiene copolymer) added to the PMMA. One study showed that the Young’s modulus and tensile strength values were reduced by increasing the rubber content [43]. Considering its high impact strength and fracture resistance, rubber-incorporated PMMA can be beneficial for patients who are prone to dropping their prosthesis repeatedly, such as senile and Parkinson’s disease patients [287]. The drawback of these materials is their high cost [75]. Currently, very little research is available regarding the chemical modification of PMMA. Therefore, further research exploring chemical modifications of PMMA using various materials is required, including cross-linkers, resins, and copolymers.

### 7.4. Antimicrobial Functionalization of PMMA

Biofilm formation and bacterial growth are always concerning due to the associated infectious diseases and financial burden on the healthcare system [288]. In the oral cavity, bacterial adhesion and plaque is associated with various prevalent conditions, including dental caries (tooth decay), periodontal diseases, and denture-induced stomatitis resulting in fungal growth [289]. Therefore, the development and functionalization of biomaterials with antimicrobial properties is always desired [288]. To impart antimicrobial properties on the materials, various modifications have been reported, such as the addition of antimicrobial polymers, inorganic nanoparticles, or medicaments, as well as surface functionalization, demonstrating promising outcomes against various oral microorganisms [290]. Several studies have reported the inhibition of microbial adhesion and growth on PMMA surfaces containing fluoride glass fillers [264,265], fluorapatite, or apatite-coated TiO_2_ [262,263]. The availability of fluoride in the oral cavity results in the enhancement of antibacterial activity against the oral microbiomes [291]. Similarly, the growth of denture-stomatitis-associated *Candida albicans* was remarkably inhibited by the addition of silver nanoparticles [257,258,259,260,261], nanodiamonds [283], and mesoporous silica nanoparticles loaded with the antifungal medicament amphotericin B [266]. The addition of thymoquinone antifungal agent (up to 1%) to the PMMA had no effect on the surface and flexural properties [292]. Quaternary ammonium compounds are also known for their antibacterial activity and have been reviewed comprehensively for the modification of various dental materials [293]. Although quaternary ammonium-based compounds showed good antimicrobial activity when added to restorative dental materials [294,295], they may affect the properties of other materials, such as the polymerization shrinkage, flexural strength, modulus, and biocompatibility, if used in higher concentrations [293]. Adding quaternary ammonium compounds (2%) to cured PMMA resulted in antibacterial and antifungal activity in vitro. Dentures containing quaternary ammonium compounds may benefit geriatric patients prone to developing denture stomatitis [296,297].

Surface functionalization is another approach that can produce antimicrobial properties and inhibition of the bacterial adhesion on a material’s surface [288]. The surface functionalization engineers the material’s surface, therefore making it less likely to affect the bulk properties. Recently, Mai et al. [298] reported on the surface functionalization of PMMA using oxygen plasma and thermal treatment. Chlorhexidine was incorporated to induce the antimicrobial activity via controlled and sustained drug release from the functionalized surface. In addition, no cellular toxicity or apoptotic cell death was observed during the cytotoxicity evaluation [298]. Lee et al. [299] incorporated graphene oxide nanosheets into PMMA and characterized for their physical and antimicrobial properties. The addition of graphene oxide nanosheets and non-thermal oxygen plasma surface treatment improved the hydrophilicity and surface antiadhesive effects [299]. Recently, various food preservatives (such as sodium metabisulfite, potassium sorbate) have been added to PMMA. The modified PMMA materials demonstrated acceptable flexural properties and enhanced antimicrobial activity without showing any cytotoxicity [241]. Although the addition of the food preservatives altered the mechanical properties, the materials still provided acceptable flexural properties.

Although the addition of antimicrobial nanoparticles has not resulted in biocompatibility issues, the majority of research has been conducted in vitro [290]. A material’s responses, including its antimicrobial activity, may be different when in a complex dynamic oral environment. Various antimicrobial additives may lead to cytotoxicity. Therefore, further in vivo clinical studies are essential to validate the efficacy of the antimicrobial agents and establish their biosafety and biocompatibility [290].

## 8. Conclusions and Future Trends

The present article comprehensively reviewed the properties, dental applications, and recent modifications of PMMA-based materials. According to ADA Specification No. 12, the majority of PMMA properties satisfactorily fulfil the requirements for denture base polymers. Nevertheless, the discoloration, hydrolytic degradation, and fracture of PMMA appliances are commonly reported in dental clinics, indicating that the properties of PMMA require further improvement. In the recent decades, plentiful research has been conducted, focusing om further improving the physical and mechanical properties of PMMA. Modifications of PMMA involving chemical or mechanical reinforcement using supplementary materials (fibers, nanofillers, nanotubes and hybrid materials) have resulted in remarkable improvements in the mechanical (impact strength, cyclic fatigue, flexural strength, and wear resistance), physical (thermal conductivity, water sorption, solubility, and dimensional stability), and biological (antimicrobial activity, biocompatibility) properties (Section 7). However, it remains challenging to improve one set of properties without compromising the rest of the properties. For example, although adding nanoparticles or fibers can improve the strength of PMMA, this may compromise the aesthetics (color, translucency) or increase the biocompatibility issues via the leaching of degradation products in the oral cavity. Although the current modifications of PMMA have resulted in encouraging outcomes, there is a long way to in using modified PMMA materials in dental clinics for practical applications. The biocompatibility and in vivo performance of modified materials are still questionable and require further investigation. Further research should focus on understanding the interactions of modified materials at the molecular levels, the evaluation of various properties following ADA specifications, and clinical performance in either simulated oral environments or in vivo clinical studies.

## Figures and Tables

**Table 1:** Chronology of the development of poly methyl methacrylate (PMMA) materials for dental applications.

Year	Development	Reference
1843	Formation of acrylic acid by oxidation of acrolein was reported	[44]
1931	Harder PMMA became commercially available in sheet forms	[45]
1936	Otto Rohm developed industrial PMMA as credit of their research in the beginning of 20th century	[46]
1937	PMMA was firstly introduced in powder form for denture base fabrication	[47]
1945	PMMA was used extensively by neurosurgeons for cranioplasties	[48]
1945	Cold-cured (room temperature curing) PMMA became commercially available	[49]
1946	PMMA became the leading material for making dentures	[47]
1950s	Orthopedic surgeon used PMMA for the cementation of femoral bones prostheses	[48]
1950s to 1960s	PMMA’s use by dental professionals dramatically increased for a variety of applications, including dentures, temporary or provisional crowns, and maxillofacial prostheses	[50]
21st century	Ongoing research and modifications of existing PMMA materials are improving the mechanical and physical properties	[51,52,53,54]

**Table 2:** Key advantages and disadvantages of various denture base materials replaced by PMMA.

Material	Advantages	Disadvantages
Gold	Gold is known for its excellent biocompatibility and corrosion resistance. Denture base was historically fabricated using gold centuries ago [59,60,61].	Expensive, poor aesthetics due to its color [60].
Porcelain	Porcelain was introduced in the 18th century for denture fabrication [60].	Very hard, high-density brittle materials with poor aesthetics [57].
Vulcanite	Vulcanite is a cost-effective material that was introduced in the 19th century and used for several years; vulcanite is dimensionally stable, comfortable, low density, light weight, and is easily fabricated [60].	Absence of chemical bonding with porcelain teeth and poor aesthetics [55].
Aluminum	Aluminum was used to cast denture bases using a casting process during the 19th century [60], providing accurate fit and light weight [57].	Casting aluminum was an expensive and sensitive technique [57].
Celluloid	A polymeric material used in the 19th century; can be colored pink to mimic oral tissues [45].	Color changes by staining from food and altered taste due to the presence of camphor [45].
Bakelite	Used in the 20th century; had excellent aesthetics [57].	Difficult manipulation, prone to staining, and brittle [57].
Polyvinyl Chloride (PVC)	A co-polymer of acetate and vinyl chloride used for dentures in the 20th century [62].	Poor mechanical properties and prone to discoloration [62].
Base metal alloys	Nickel and cobalt chromium alloys have been used since early 20th century. Still used due to their excellent mechanical properties, low density, and cost-effectiveness [57].	Poor aesthetics due to metallic color, very hard materials; difficult to cut, finish, and repair. There are allergy issues mainly due to the presence of nickel [57].

**Table 3:** Various heat cycles used for the polymerization of heat-cured PMMA.

Heat Cycles	Temperature	Time	Terminal Boiling	Reference
1	74 °C	8 h	None	[78]
2	74 °C	8 h	1 h	[78]
3	74°C	3 h	1 h	[78]
4	73.9 °C	12 h	None	[50]
5	70 °C	3 h	1 h	[83]
6	100 °C	20 min	None	[84]

**Table 4:** Various properties of heat-cured PMMA.

Property	Value	Reference
Elastic modulus (GPa)	2.6	[7]
	3.89 ± 1.320	[98]
Flexural strength (MPa)	90	[7]
Fracture toughness (MN/m^3/2^)	2.53	[7]
Fracture toughness (MPa.m^1^^/^^2^)	1.86 ± 0.25	[66]
Proportional limit (MPa)	26	[82]
Compressive strength (MPa)	76	[82]
Tensile strength (MPa)	48–62	[82]
	55	[7]
Elongation (%)	1–2%	[82]
Impact strength (J)	0.98–1.27 J	[66,82]
Knoop hardness (KHN)	18–20 KHN	[82]
Rockwell hardness	M90–M100	[107,108]
Vickers hardness (VHN)	20	[7]
Absolute hardness (MPa)	297.72 ± 19.04	[98]
Fatigue strength (MPa)	1.5 × 10^6^ cycles at 17.2 MPa	[82]
Biaxial flexural strength	121 ± 12	[66]
Thermal conductivity	5.7 × 10^−4^ °C/cm	[82]
Coefficient of thermal expansion	81 × 10^−6^ /°C	[82]
Linear thermal expansion (mm/mm.k)	6.3 × 10^−5^	[108]
Glass transition temperature	125 °C	[7,82]
Curing shrinkage (%)	−0.50 to −0.58	[95]
Density g/cm^3^ at room temperature	1.18	[107,108]
Sorption (mg/cm^2^)	0.69	[82]
Solubility (mg/cm^2^)	0.02 (water), 0.04 (hydrocarbons)	[82]
Color	Transparent, colorless	[7]

**Table 5:** Comparison of conventional heat-cured and CAD/CAM PMMA properties.

Property	Conventional Heat-Cured PMMA	CAD/CAM PMMA	Reference
Chemistry	Similar		[208]
Monomer leaching	No significant differences		[212]
Biocompatibility	Similar		[208]
Manipulation	Flask-pack-press’	Rapid prototyping and milling techniques	[208]
*Candida albicans* adherence/stomatitis		Reduced	[207]
Mechanical properties		Improved	[204,208]
Hardness		Increased	[206]
Flexural strength		Improved	[203,204,205]
Flexural modulus and impact strength		Improved	[204]
Durability		Improved	[204]
Teeth bond strength	Higher; reduced with aging	Lower; less effect with aging	[209]
Hydrophobicity		Higher	[205,206,207]
Contact angle	Lower		[205]
Surface roughness	Similar		[205]
		Lower	[207]
		Ra=0.2µm, which is below the threshold for plaque accumulation	[123]

**Table 6:** Characteristics of various fibers used for the mechanical reinforcement of PMMA.

Fiber type	Characteristic Description and Main Outcomes	Reference
Carbon	Enhancement of the mechanical properties, including tensile strength, flexibility, fracture resistance, and elastic modulus.	[20,242]
	Reduced thermal expansion of modified PMMA materials.	[20]
	Poor aesthetic properties due to the color of fibers.	
Aramid (Kevlar)	Polyamide fibers that have better wettability (coupling pre-treatment is not required) and improved mechanical properties, such as fracture resistance.	[243]
	Poor aesthetics due to yellowish color; fibers exposed to the surface are irritable to patient’s tissues. Difficult to finish and polish the surface.	[18,75,244]
	Increasing the concentration of fibers reduced the hardness.	[18,75]
Nylon	Adding nylon fibers improved the flexural strength.	[245]
	Improved structural elasticity and fracture resistance.	[103]
Polyethene and polypropylene	Adding surface-treated fibers improved the impact strength.	[21,139]
	Superior toughness and ductility.	[22,246,247]
	In addition to impact strength, adding silanized fibers improved the tensile and transverse strengths of heat-cured PMMA, however wear resistance was poor.	[17]
	The aesthetic properties were not affected due to their white color.	[21,243]
	Technique was sensitive and required surface treatment, therefore is not used extensively.	[243]
Glass	Glass fibers can be used in various forms (woven, loose); provide excellent reinforcement and aesthetics compared to other fibers.	[245]
	A remarkable increase in the denture base toughness, Vickers hardness, impact strength, and flexural strength was observed.	[15,16,248,249]
	A remarkable reduction in the deformation (<1%).	[15]
	The silanized glass fibers enhanced the flexural strength, while the strength of modified PMMA may be influenced by the proportion and positioning of fibers.	[19]

**Table 7:** Characteristics of various filler particles used for the mechanical reinforcement of PMMA.

Particles	Modification and Outcome	Reference
Alumina (Al_2_O_3_)	Addition of alumina Al_2_O_3_ nanoparticles to PMMA powder resulted in good biocompatibility.	[277]
	Silane-treated aluminium particles remarkably improved the mechanical properties—mainly the compressive and flexural strengths, as well as the wear resistance.	[25,26]
	No significant effects on the water sorption or surface roughness of PMMA.	[145]
	Significantly improves the thermal conductivity of PMMA.	[255]
	The main limitation of the Al_2_O_3_ reinforcement is that it causes discoloration of the resin.	[25]
Zirconia (ZrO_2_)	Significant improvement in mechanical properties, including fracture toughness, compressive and fatigue strengths.	[29,30,31,32]
	Using silane coupling agent (3.5%) improved the PMMA–ZrO_2_ interface and flexural strength.	[278]
	ZrO_2_ nanoparticles (NPs) added to the PMMA improved the thermal conductivity.	[31]
	The ZrO_2_ nanotubes demonstrated superior reinforcing effects compared to ZrO_2_ particles.	[270]
	Increased the water sorption, however it remained within the limits.	[279]
Titania (TiO_2_)	Adding TiO_2_ particles enhanced the thermal conductivity, fracture toughness, and hardness.	[254]
	Increase in the impact strength.	[280]
	The addition of silanized TiO_2_ particles showed similar effects by improving the surface hardness, transverse and impact strengths. The water sorption and solubility were also reduced.	[267]
	The fluorapatite or apatite-coated TiO_2_ demonstrated antifungal effects and inhibited *Candida* growth.	[262,263]
	Due to poor wettability, there is a need for titanium coupling agent reinforcement in PMMA. The modification of PMMA by the incorporation of barium titanate (radiopacifier) reduced the fracture toughness.	[281]
Silver	Due to their metallic nature, adding silver particles improved the compressive strength and thermal conductivity of PMMA.	[23,24]
	Due to their antimicrobial properties, silver particles inhibit bacterial attachment. Dentures containing siliver nanoparticles have shown antifungal activity.	[257,258,259,260,261]
	The addition of silver and graphene nanoparticles to PMMA significantly enhanced the mechanical properties (tensile, compressive, and flexural strengths) and lowered the water absorption.	[268]
	No significant changes in the flexural strength of PMMA.	[255]
Nanodiamond (ND)	The ND particles are bioactive and reinforce acrylic polymers.	[54,282]
	Adding ND nanoclusters (20nm; ~0.83 wt%) to PMMA significantly improved the elastic modulus (~2.084GPa) and impact strength.	[282]
	Adding only 0.1 wt.% ND to PMMA remarkably increased the flexural strength, in addition to inhibiting the growth of *Candida albicans* fungal infection and salivary biofilm.	[283]
	Diminished *Candida albicans* attachment corresponding to the reduced surface roughness, therefore may benefit in the prevention of denture stomatitis.	[284]
Hydroxyapatite (HA)	Inorganic HA improved the PMMA properties, including the elastic modulus and flexural strength.	[27,28]
Silica (SiO_2_) based particles	The SiO_2_ nanoparticles improved the mechanical properties of PMMA.	[33,34,35,36]
	The PMMA modified by mica showed improved surface hardness, dimensional stability, and thermal properties. However, the flexural strength was compromised due to the weakening of mica bonding with the acrylic resin.	[256]
	The fluoride glass fillers containing PMMA inhibited the microbial adhesion but enhanced the surface roughness.	[264,265]
	The incorporation of mesoporous silica nanoparticles loaded with an antifungal drug (amphotericin B) resulted in long-term antifungal activity against *Candida albicans*.	[266]
	Increasing the concentration of nanosilica may lead to biocompatibility issues; however, at lower concentrations (less than 2%) there is no cytotoxicity.	[35]
